# From Bariatric Surgery to Conception: The Ideal Timing to Optimize Fetal Weight

**DOI:** 10.1007/s11695-023-06755-4

**Published:** 2023-07-22

**Authors:** Ana Carreira, Bárbara Araújo, Mariana Lavrador, Inês Vieira, Dírcea Rodrigues, Sandra Paiva, Miguel Melo, Isabel Paiva

**Affiliations:** 1 Department of Endocrinology, Diabetes and Metabolism, Centro Hospitalar E Universitário de Coimbra, 3004-561 Coimbra, Portugal; 2 Faculty of Medicine, University of Coimbra, 3000-548 Coimbra, Portugal

**Keywords:** Bariatric surgery, Small for gestational age, Pregnancy, Neonatal, Weight, Counselling

## Abstract

**Purpose:**

Bariatric surgery (BS) increases the risk of small for gestational age (SGA) neonates. Guidelines recommend postponing pregnancy for 12–24 months, but optimal surgery-to-conception interval (BSCI) remains uncertain. We aimed to evaluate the impact of BSCI on birth weight and SGA.

**Materials and Methods:**

Retrospective cohort study of 42 pregnancies following BS, including Roux-en-Y gastric bypass, gastric sleeve, adjustable gastric banding and biliopancreatic diversion. Neonates were classified as SGA if birth weight < 10^th^ percentile. Optimal BSCI was obtained from the analysis of ROC curves, and pregnancies were compared by that cut-off.

**Results:**

There was a linear association between BSCI and birth weight and an inverse association with SGA, with each additional month of BSCI translating into additional 4.5 g (95%CI: 2.0–7.0) on birth weight and -6% risk of SGA (95%CI: 0.90–0.99). We established a cut-off of 24.5 months of BSCI for lower risk of SGA. Pregnancies conceived in the first 24 months had a more than tenfold increased risk of SGA (OR 12.6, 95%CI: 2.4–66.0), even when adjusted for maternal age, gestational diabetes and inadequate gestational weight gain.

**Conclusion:**

BSCI was associated with birth weight and SGA. Our results are in line with the recommendations of BSCI of at least 24 months to reduce the risk of SGA.

**Graphical Abstract:**

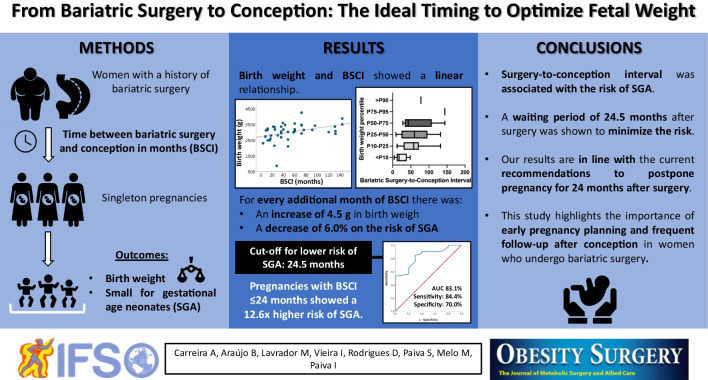

## Introduction

Obesity is a major public health problem and its prevalence is increasing steadily among women of reproductive age [1]. Obesity is associated with a higher prevalence of infertility and negative results following assisted reproduction treatments [2], as well as a higher risk of adverse maternal and fetal outcomes in pregnancy, including gestational diabetes, preeclampsia, cesarean delivery, preterm delivery, large size for gestational age, and infant death [3, 4]. Bariatric surgery (BS) is the most effective treatment for severe obesity, and more than half of all bariatric procedures are currently performed on women of reproductive age [5, 6]. BS has been shown to improve fertility and reduce obesity-related complications in pregnancy [5–7]. However, pregnancies after BS have been associated with lower birth weight and with twice the risk of small for gestational age (SGA) neonates when compared with pregnancies with the same pre-gestational body mass index (BMI) [6, 8, 9]. SGA have a higher risk of perinatal morbidity and mortality [10], along with a higher risk of insulin resistance, obesity, cardiovascular disease and type 2 diabetes mellitus later in life [11]. Immediately after BS, patients undergo a catabolic phase, with marked caloric restriction, metabolic derangement and rapid weight loss, which may impair adequate fetal development in pregnancies conceived shortly after surgery [14, 20]. Studies have associated several factors with increased risk of SGA in pregnancies following BS, with recent emphasis on nutrient deficiencies and inadequate gestational weight gain (GWG) [9, 12]. Some studies additionally suggested that pregnancies conceived shortly after surgery are at greater risk of SGA [13, 14]. Still, the impact of bariatric surgery-to-conception interval (BSCI) on the risk of SGA remains uncertain, as different studies have conflicting findings and most consist of small cohorts [6]. It is known that the greatest weight loss usually occurs in the first 12–24 months after BS, depending on the procedure type [15, 16]. One study evaluating weight dynamics at the onset of pregnancy showed that most women who conceived more than 18 months after BS achieved weight stabilization, in contrast with those who conceived earlier than 18 months, where nearly half were still losing weight [17]. Thus, current guidelines advise against conception in the first 12 months after BS [18] and most international societies recommend delaying pregnancy for 12–18 months [19], or 12–24 months [20] to ensure weigh stabilisation and correction of nutrient deficiencies before pregnancy. A recent consensus suggests individualizing BSCI according to time until weigh stabilization: typically, 12 months after gastric sleeve or gastric bypass and 24 months after adjustable gastric banding (AGB) [15]. However, the evidence on which these recommendations are based is limited and optimal BSCI is yet to be determined.

Therefore, our purpose in this study was to evaluate the impact of BSCI on birth weight and on the risk of SGA. Additionally, we aimed to determine the ideal BSCI to reduce the risk of SGA in our population.

## Methods

### Study Design and Participants

We performed a retrospective cohort study of singleton pregnancies of female patients with a history of BS, with follow-up in a University Hospital Centre in Portugal, between 2008 and 2020. Cases of twin pregnancies, spontaneous abortion, voluntary termination of pregnancy or intrauterine fetal demise were excluded. Term pregnancies with a follow-up that did not include the three trimesters of pregnancy and pregnancies without birth data were also excluded. BS procedures included AGB, sleeve gastrectomy, Roux-en-Y gastric bypass and biliopancreatic diversion and were performed between 2005 and 2018. Data regarding demographics, comorbidities, type of BS, date of BS, BMI previous to surgery and previous to conception, GWG and pregnancy outcomes were collected from electronic health records. GWG was calculated as the difference between weight at the time of delivery and self-reported pre-gestational weight. Weight gain was classified as inadequate, adequate or excessive based on the pre-gestational BMI, according to the 2009 Institute of Medicine (IOM) recommendations. Thus, adequate weight gain was defined as a total weight gain of 11.5–16.0 kg in women with normal weight (BMI 18.5–24.9 kg/m^2^), 7.0–11.5 kg in overweight (BMI 25.0–29.9 kg/m^2^) and 5.0–9.0 kg in women with obesity (BMI ≥ 30 kg/m^2^) [21].

The study was approved by the local ethical committee of our hospital.

### Exposures and Outcomes

The exposure of interest was the time between BS and conception. This interval was calculated in months from the date of surgery to the approximate date of conception. The date of conception was defined as that estimated in the first trimester ultrasound.

Our primary outcomes were birth weight and SGA status. Neonates were classified as SGA if birth weight was inferior to the 10^th^ percentile and as large for gestational age (LGA) if above the 90^th^ percentile, according to the World Health Organization (WHO) growth charts in term births, or Fenton curves in preterm births. Preterm births were defined according to WHO, as births occurring before 37 completed weeks of gestation, and classified as extremely preterm when occurring before 28 weeks, very preterm between 28 and 32 weeks and moderate to late preterm between 32 and 37 weeks.

### Statistical Analysis

Analyses were performed with the use of IBM SPSS Statistics 26.0. Categorical variables are presented as frequencies and percentages, and continuous variables as means and standard deviations, or medians and interquartile ranges for variables with skewed distributions. The means or medians of continuous variables were compared between patient groups using the Student’s T-test for independent samples or the Mann Whitney test, respectively. Associations between categorical variables were assessed using the Chi-square test. A cut-off value of BSCI for reducing the risk of SGA was obtained from the analysis of ROC curves. Pregnancies were categorized in two groups according to the obtained cut-off of BSCI, and multiple logistic regression was used to compare the odd of SGA in both groups and control for possible confounding factors. Covariates were selected based on their known clinical impact on fetal weight. Multiple linear regression was used to assess the impact of BSCI on birth weight adjusted to gestational age. All reported *P* values are two-tailed, with a *P* value of less than 0.05 indicating statistical significance.

## Results

From a total of 47 singleton pregnancies with complete follow-up, four (8.5%) resulted in spontaneous abortion and one (2.1%) in fetal demise at 30 weeks. Excluding these, we obtained a total of 42 pregnancies that lead to 39 term births and 3 preterm births: 1 extremely preterm and 2 moderate to late preterm. As presented in Table 1, the most common BS procedures were Roux-en-Y gastric bypass (40.5%) and sleeve gastrectomy (38.1%), followed by AGB (16.7%) and biliopancreatic diversion (4.8%). All patients were followed by an endocrinologist and received individualized supplementation with folic acid, iron, calcium and vitamin D according to measured analytes; 77.5% were also supplemented with multivitamins (68.2% of patients with restrictive procedures and 88.9% of patients with malabsorptive procedures). Mean maternal age was 34.3 ± 4.5 years and mean BMI at conception was 30.0 ± 5.5 kg/m^2^. Mean BMI reduction from surgery to conception was 14.9 ± 7.4 kg/m^2^; leading to a normal BMI at conception in 21.4% of the women, overweight in 28.6% and obesity in 50.0% (33.3% class I, 11.9% class II and 4.8% class III). BSCI ranged from a minimum of 4 to a maximum of 144 months. Six pregnancies (14.3%) were conceived on the first 12 months after BS, six (14.3%) between 12 and 24 months and 30 (71.4%) after more than 24 months. Mean fetal birth weight was 3103 g (interquartile range [IQR] 2780-3361 g); 10 neonates were SGA (28.3%) and one was LGA (2.4%). All SGA neonates were born at term, with median gestational age of 38 weeks (IQR 38–39 weeks) and mean birth weight of 2498 ± 233 g. Most neonates (87.5%) had birth weight inferior to the 50^th^ percentile (P): 28.3% inferior to P10, 19.0% between P10-P25 and 40.5% between P25-P50; 11.9% had birth weight between P50-P75, 2.4% between P75-P90 and 2.4% above P90. There was no association between SGA and bariatric procedure type, and there was no significant difference in maternal age or pre-gestational BMI in SGA and non-SGA neonates. GWG was adequate in 30.0% of all pregnancies, inadequate in 32.5% and excessive in 37.5%. The prevalence of SGA was 30.8% in pregnancies with inadequate GWG versus 8.3% in pregnancies with adequate GWG, but this difference did not reach statistical significance (*p* = 0.70).

**Table 1:** Baseline characteristics of pregnant women and comparison between pregnancies with bariatric surgery-to-conception intervals up to 24 months and over 24 months

Characteristic	All (*N* = 42)	BSCI ≤ 24 months (*N* = 12)	BSCI > 24 months (*N* = 30)	*P* value
Prepregnancy characteristics				
Maternal age (years), mean ± SD	34.2 ± 4.4	34.9 ± 4.4	34.0 ± 4.5	0.538
BS procedure, n (%)				
AGB	7 (16.7)	1 (8.3)	6 (20.0)	0.651
SG	16 (38.1)	6 (50.0)	10 (33.3)	0.483
RYGB	17 (40.5)	4 (33.3)	13 (43.3)	0.731
BPD	2 (4.8)	1 (8.3)	1 (3.3)	0.495
BMI before BS (kg/m^2^), mean ± SD	44.9 ± 5.8	45.4 ± 5.7	44.8 ± 6.0	0.758
BSCI (months), mean ± SD	53.9 ± 40.3	14.8 ± 6.5	69.5 ± 37.4	< 0.001
BMI at conception (kg/m^2^), mean ± SD	30.0 ± 5.5	30.9 ± 5.1	29.6 ± 5.7	0.494
Hypertension, n (%)	2 (4.8)	1 (8.3)	1 (3.3)	0.495
History of spontaneous abortion/fetal demise, n (%)	11 (26.2)	2 (16.7)	9 (30.0)	0.456
First pregnancy, n (%)	13 (31.0)	6 (50.0)	7 (23.3)	0.141
Maternal and fetal outcomes				
GWG (kg), mean ± SD^a^	9.1 ± 8.3	9.1 ± 9.7	9.1 ± 7.9	0.990
Inadequate^b^, n (%)	13 (32.5)	5 (41.7)	8 (28.6)	0.455
Adequate^b^, n (%)	12 (30.0)	0 (0.0)	12 (42.9)	0.007
Excessive^b^, n (%)	15 (37.5)	7 (58.3)	8 (28.6)	0.153
Gestational diabetes, n (%)	8 (19.0)	4 (33.3)	4 (13.3)	0.195
Pre-eclampsia, n (%)	1 (2.4)	1 (8.3)	0 (0.0)	0.286
Preterm birth, n (%)	3 (7.1)	0 (0.0)	3 (10.0)	0.545
Cesarean delivery, n (%)	13 (31.0)	4 (33.3)	9 (30.0)	1.000
Gestational age (weeks), median (IQR)	39.0 (38.0–40.0)	39.0 (38.0–39.8)	39.0 (38.0–40.0)	0.611
Neonate male gender, n (%)	19 (45.2)	6 (50.0)	13 (43.3)	0.695
Birth weight (g), median (IQR)	3103 (2780–3361)	2765 (2500–3214)	3150 (2945–3455)	0.037
SGA, n (%)	10 (23.8)	7 (58.3)	3 (10.0)	0.002
LGA, n (%)	1 (2.4)	0 (0.0)	1 (3.3)	1.000

*BSCI*: bariatric surgery-to-conception interval; *BMI*: body mass index; *BS*: bariatric surgery, *AGB*: adjustable gastric banding; *SG*: sleeve gastrectomy; *RYGP*: Roux-en-Y gastric bypass; *BPD*: biliopancreatic diversion; *IQR*: interquartile Range; *SD*: standard deviation; *GWG*: gestational weight gain; *SGA*: small for gestational age, *LGA*: large for gestational age. ^a^Two missing values for GWG. ^b^Characterized according to the 2009 IOM recommendations

### Bariatric Surgery-to-Conception Interval, Fetal Weight and SGA

**Fig. 1:**
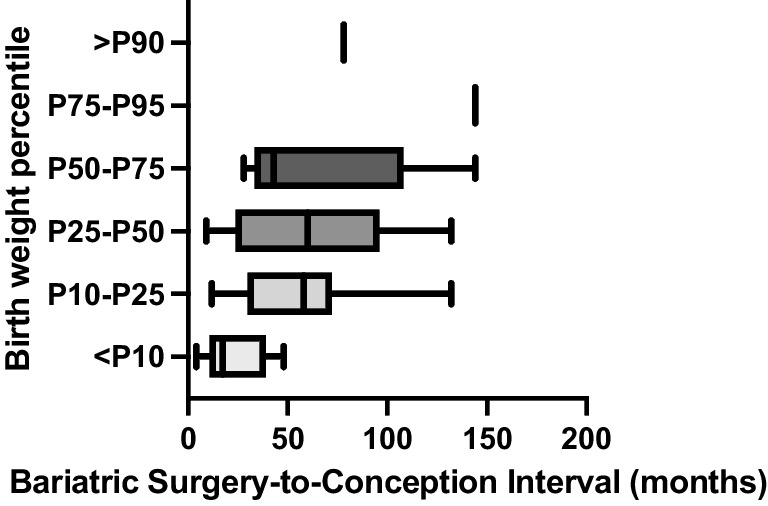
Distribution of bariatric surgery-to-conception interval (BCSI) means across neonate birth weight percentiles. P: percentile. Mean BSCI was 23.1 ± 15.8, 56.0 ± 36.1, 61.3 ± 41.0 and 65.2 ± 47.0 months for birth weight percentiles < P10, P10-P25, P25-P50 and P50-P75, respectively. There was only one neonate with birth weight between P75-P90 and one with birth weight > P90

BSCI showed a linear relationship with birth weight. By multiple linear regression, we estimated a birth weight increase of 4.5 g for each month of BSCI (95% CI 2.0–7.0), adjusting for gestational age (R^2^_a_ = 66.1%). Comparing BSCI across birth weight percentiles there was a similar association, with progressively higher birth weight percentiles (< P10 to P75) showing progressively longer mean BSCI (Fig. 1). This difference was more pronounced for neonates with birth weight < P10. Accordingly, mean BSCI was significantly shorter in pregnancies with SGA (23.1 ± 15.8 vs 63.5 ± 40.9 months, *p* < 0.001). Logistic regression confirmed an inverse association between BSCI and SGA, in which for every additional month of BSCI there was a decrease of 6% on the risk of SGA (OR 0.94, 95% CI 0.90–0.99).

**Fig. 2:**
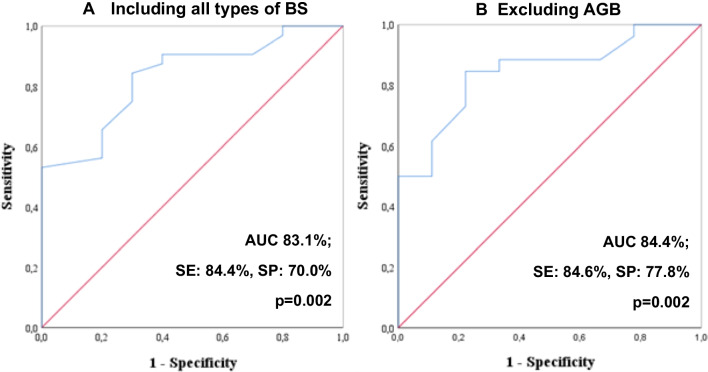
Roc curves analysis to establish a minimum cut-off of time between bariatric surgery and conception associated with lower risk of SGA neonates. BS: bariatric surgery; AGB: adjustable gastric banding; AUC: area under the curve; SE: sensitivity; SP: specificity. AGB was excluded from the analysis to evaluate if there was a lower cut-off of BSCI in the remaining BS types, as suggested by some expert consensus; however, the obtained cut-off was the same

By the analysis of ROC curves, we established a cut-off of a minimum of 24.5 months of BSCI for lower risk of SGA neonates (sensitivity: 84.4%, specificity: 70.0%) [Fig. 2A]. The prevalence of SGA neonates was similar across the different types of BS. Given the fact that some consensus recommend a BSCI of more than 24 months only after AGB and of more than 12 months after gastric sleeve or gastric bypass, we performed the same analysis after excluding the cases of previous AGB surgery, and obtained the same cut-off value of 24.5 months (sensitivity: 84.6%, specificity: 77.8%) [Fig. 2B]. Categorizing BSCI according to this cut-off, as displayed in Table 1, we obtained a prevalence of SGA of 58.3% and median birth weight of 2765 g (IQR 2500-3214 g) in the group of BSCI of up to 24 months, contrasting to a prevalence of SGA of 10.0% and median birth weight of 3150 g (IQR 2945-3455 g) in the group of BSCI of more than 24 months. In multiple logistic regression analysis (Table 2), the risk for SGA increased more than tenfold in pregnancies with BSCI of less than 24 months (OR 12.6, 95% CI 2.4–66.0), even when adjusted for inadequate GWG, maternal age and gestational diabetes (OR 22.1, 95% CI 3.0–162.3). Pregnancies with inadequate GWG did not show significantly increased odds for SGA in univariate (*p* = 0.389) or multivariate analysis adjusted for BSCI (*p* = 0.501). Excluding the 6 pregnancies that were conceived on the first 12 months after BS, the association between BSCI and SGA remained significant, with a higher prevalence of SGA in those who conceived between 12 and 24 months after surgery, when compared to those who conceived after 24 months (66.7% vs 10.0%, *p* = 0.008).

**Table 2:** Multiple logistic regression model to assess the impact of bariatric surgery-to-conception interval on the risk of small for gestational age neonates

Small for gestational age neonate						
Variable	Crude OR	95% CI	*p* value	Adjusted^a^ OR	95% CI	*p* value
BSCI ≤ 24 months	12.6	2.4–66.0	0.003	22.1	3.0–162.3	0.002
BSCI (months)	0.94	0.90–0.99	0.016	0.93	0.88–0.99	0.014

*CI* – Confidence Interval; *OR* – Odds Ratio. *BSCI* – Bariatric surgery-to-conception-interval. ^a^Adjusted for: maternal age, gestational diabetes and inadequate gestational weight gain

There was no correlation between BSCI and BMI reduction from surgery to conception and BMI reduction was similar in pregnancies with BSCI of up to 24 months or longer than 24 months. Additionally, pregnancies conceived after the first 24 months following BS showed a greater prevalence of adequate GWG (42.9% vs 0.0%) and no significant difference in the prevalence of excessive GWG (28.6% vs 58.3%, *p* = 0.153).

## Discussion and Conclusion

In this study, we show an association between time from BS to conception and birth weight and risk of SGA. More than a quarter of neonates were SGA and the vast majority had a birth weight percentile of less than 50. BSCI showed a linear association with birth weight and an inverse association with the risk of SGA. Furthermore, we obtained an optimal cut-off of at least 24.5 months from BS to conception to reduce the risk of SGA, independently of BS type. Women who conceived in the first 24 months after BS had a high prevalence of both inadequate and excessive GWG, and displayed a 13 times higher risk of having an SGA neonate.

As described in previous studies, despite significant weight loss, the prevalence of obesity remained elevated among women who underwent BS [13, 22], but the prevalence of obesity-related pregnancy complications such as LGA was reduced. The prevalence of SGA in our study was more than double of the prevalence of the general population in Portugal and Europe [23]; and in line with evidence reported in other European studies of women submitted to BS [9, 17]. The low prevalence of adequate weigh gain was also consistent with other studies [13]. Current evidence on the impact of BSCI on birth weight and SGA is conflicting, as most studies have important limitations in sample size and heterogeneity in the type of BS. Also, most studies divide pregnancies by BSCI of up to 12 months or longer [24]; and, to date, few studies have analysed the difference between BSCI of up to 24 months or longer. In our analysis, SGA was more prevalent in BSCI of up to 24 months than in the remaining pregnancies, even after excluding pregnancies conceived before 12 months. Therefore, the threshold of 12 months to compare the risk of SGA may be misleading, especially if the analysis does not include an adequate number of considerably longer intervals. Among the studies that analysed longer intervals from BS to conception, we found some results similar to ours: *Parent *et al. analysed a cohort of 1859 pregnancies and reported a higher risk of SGA in those conceived within the first 2 or 2–4 years after BS, compared to those conceived after more than 4 years [14], *Rasteiro *et al. described a lower fetal growth percentile in pregnancies conceived before 24 months [25], and *Heusschen *et al. showed a progressive increase in birth weight and decrease in SGA prevalence from BSCI of less than 12 months to BSCI of more than 24 months [13]. Regarding GWG, our results showed a lower prevalence of adequate weight gain in pregnancies conceived after a shorter BSCI and a higher prevalence of SGA in the presence of inadequate weight gain, also in agreement with the findings of *Heusschen *et al. [13]. Additionally, and unlike other studies [22], we did not find a significant difference in excessive weight gain or in BMI reduction from surgery to conception in pregnancies conceived later after BS, and there was a similar prevalence of excessive weight gain in pregnancies conceived earlier than 24 months. A possible explanation for this is that shorter intervals between BS and pregnancy may lead to inadequate weight gain due to marked catabolism and caloric restriction, but also to excessive weight gain due to the interruption of weight loss before maximal loss is achieved. Concerning the association between BSCI and fetal weight, we hypothesize that a shorter time between BS and conception may be a reflection of a set of many harmful exposures to the fetus that go beyond inadequate GWG, such as a negative energy balance and macronutrient and micronutrient deficiencies. This hypothesis and the obtained cut-off for BSCI are also in line with the evidence that weight loss and metabolic derangements are more pronounced in the first 12–24 months after BS. However, it requires validation by prospective studies analysing patients’ weight loss curves, micronutrient deficiencies and protein-energy status from BS to conception and during pregnancy. Still, our results support current guidelines that suggest an optimal delay of more than 12–24 months between BS and conception in order to optimize fetal weight. As obesity and infertility rise among women of reproductive age, it becomes crucial that women with obesity are aware of the risks of early conception after BS, and, ideally, BS should be programmed in advance, in order to allow adequate pregnancy planning with a two-year interval. Nonetheless, we agree with the consensus recommendations [15, 18], that suggest that the ideal BSCI can be individualised in each patient, and shorter intervals may be considered for women who have stabilized weight and no significant nutritional deficiencies. Adequate follow-up of these patients from BS to pre-conception and during conception is, therefore, of the utmost importance.

Our study has several strengths. We analysed a cohort that included several pregnancies with considerably long intervals from BS to conception; the characteristics of pregnancies conceived before and after the first 24 months following BS were homogeneous with respect to maternal age, surgery type, BMI reduction and pre-gestational BMI. We proposed an “ideal cut-off” for BSCI, with elevated sensitivity and specificity, in order to minimize the risk of having an SGA neonate; the obtained cut-off is in line with current recommendations.

However, our study has several important limitations. It was a retrospective analysis, which relied on the availability of clinical registries. Data on nutritional deficiencies were limited and, therefore, not included in the analysis, which represents a possible confounding factor. GWG was heterogeneous between BSCI groups, with pregnancies conceived before 24 months showing a higher prevalence of inadequate GWG. Additionally, pregnancies with inadequate GWG had more SGA. Thus, although we did not find a statistically significant impact of inadequate GWG in SGA, the limited sample size does not allow us to overrule a confounding effect of GWG in our study. Also, our analysis does not allow to discriminate between pathological and constitutional SGA, as ultrasound assessment of fetal growth during pregnancy was not investigated, neither did we use customized charts that adjust birth weight for parent’s characteristics. Different types of BS were included, but with small sample size. Moreover, given the limited sample size and small number of pregnancies with a BSCI of less than 24 months, the study has insufficient statistical power to allow the generalization of the obtained results and recommendations. Therefore, we emphasize the need for larger studies, with a sufficient number of pregnancies within each type of BS and within each BSCI interval category, in order to confirm the ideal cut-off of time between surgery and conception.

In conclusion, our results support the association of shorter BSCI with lower birth weight and increased risk of SGA, and suggest delaying conception for 24 months to minimize this risk for most women. However, we recognize that frequent follow-up and monitoring may allow for the individualization of BSCI in each woman.

## Data Availability

The data that support the findings of this study are available from the corresponding author upon reasonable request.
